# Noninvasive brain stimulation of addiction: one target for all?

**DOI:** 10.1093/psyrad/kkab016

**Published:** 2021-12-14

**Authors:** Qingming Liu, Tifei Yuan

**Affiliations:** Center for Brain, Mind and Education, Shaoxing University, Shaoxing 312000, China; School of Teacher Education, Shaoxing University, Shaoxing 312000, China; Shanghai Key Laboratory of Psychotic Disorders, Shanghai Mental Health Center, Shanghai Jiao Tong University School of Medicine, Shanghai 210109, China; Co-innovation Center of Neuroregeneration, Nantong University, Nantong, Jiangsu 226019, China

**Keywords:** noninvasive brain stimulation, repetitive transcranial magnetic stimulation, transcranial direct current stimulation, dorsolateral prefrontal cortex, addiction

## Abstract

Noninvasive brain stimulation includes repetitive transcranial magnetic stimulation (rTMS) and transcranial direct current stimulation (tDCS), and emerges as a prospective approach for addiction treatment in clinical practices. The dorsolateral prefrontal cortex (DLPFC) is regarded as the most effective stimulation target, giving its important position in controlling cue-elicited drug craving and initiating drug abuse. In this paper, through literature searches (e.g. Pubmed, Google Scholar), 34 studies (2003–2021) were identified examining the effect of rTMS, tDCS on cravings, and consumption of substance use disorders, including tobacco, alcohol, opioids, and stimulants. We summarize the main methods, designs, and effects of rTMS or tDCS that are delivered to the DLPFC on different types of addiction. We conclude that targeting DLPFC might be effective for all types of drug addiction.

## Introduction

Addiction impacts public health significantly, with a high relapse rate in the current set of management. In past decades, noninvasive brain stimulation (NIBS), including repetitive transcranial magnetic stimulation (rTMS) and transcranial direct current stimulation (tDCS), is demonstrating their efficiency and advantages as alternative approaches in addiction treatment. The basic principle of brain stimulation-based therapy is to restore the physiological synaptic strength and to rehabilitate the neural circuits impaired in disease.

Currently, there are multiple protocols for clinical studies using rTMS to treat substance use disorders. The lack of protocol consistency in such rTMS studies is common, as there is no systematic approach to elucidate which parameters can best achieve specific goals. Nonetheless, there is general consensus among researchers regarding the cerebral cortex to be stimulated: targeting the modulatory midbrain cortical system, most studies have targeted the DLPFC, and only a few have stimulated the medial prefrontal cortex (mPFC) (Hanlon *et al*., 2017). Furthermore, most studies tend to stimulate only one side of the brain, usually the left side, although some studies comparing stimulation of the DLPFC on the left and right side of the brain have not shown significant brain lateralization effects (Liu *et al*., 2017). Addiction leads to systemic changes in the mesolimbic dopamine system and connected brain regions, including nucleus accumbens, amygdala, the ventral tegmental area, prefrontal cortex, and hippocampus (Hou *et al*., 2014). Within all these areas, the dorsolateral prefrontal cortex (DLPFC) direct or indirect projects to and controls the functioning of rest regions, and is controlling cue-elicited drug cravings. In terms of manipulation, targeting DLPFC has operational convenience due to the shallow location compared to other regions (Luigjes *et al*., 2019). Indeed, NIBS-mediated functional modulations on the DLPFC affect the release of neurotransmitters, such as the dopamine efflux in different brain regions (Spagnolo and Goldman, 2017), or altered response inhibition in addicts (Goldstein and Volkow, 2011). DLPFC itself is involved in emotion regulation, cognitive control, and decision-making, all of which were implicated in addiction (Volkow and Baler, 2014). All these pieces of evidence emphasized the potential importance of DLPFC in multiple aspects in addiction rehabilitation.

Here we summarize the effects of noninvasive stimulation targeting DLPFC in the context of addiction medicine. For the literature search, “transcranial magnetic stimulation” and “addiction” or “transcranial direct stimulation” and “addiction” was input on PubMed or Google scholar from 2003 to 2021.

## The Basic Principles and Applications in Addiction of tDCS and rTMS

Both tDCS and TMS could modulate cerebral cortex function in two directions: excitation and inhibition (Jansen *et al*., 2013). Through saline-soaked electrode pads, tDCS sends a constant direct current to the cortex. Some parts of the current spread along the scalp and the others penetrate the skull into the brain, which causes intracranial depolarization/hyperpolarization in a safe, efficient, and painless way. Previous research has demonstrated that this type of current effectively regulates the excitability of the cortical circuit (Nitsche and Paulus, 2001). The anode stimulation increases resting potential depolarization and may increase the frequency of spontaneous discharge of neurons, which leads to increased neuronal excitability, while the cathode stimulation induces the resting potential hyperpolarization and reduces the excitability of neurons.

The principle of TMS is to place a coil over the skull, and pass through alternating short current pulses, which generate alternating magnetic fields that induce electrical pulses in the brain (George and Aston-Jones, 2010). The induced current within the cortex is therefore determined by the frequency, strength, direction, and area of the current passing through the coil. Generally speaking, low-frequency (LF ≤ 1 Hz) TMS decreases neuronal activity and cortical excitability, and high-frequency (HF ≥ 5 Hz) TMS evokes a contrary outcome (Hallett, 2007).

An increasing number of tDCS and TMS studies have been performed for treating addictive disorders. Despite the design differences (e.g. duration of sessions, interval time, number, and sample size), most of the studies have adopted targeted stimulation to the DLPFC for the purpose of reducing cue-evoked craving (Spagnolo and Goldman, 2017).

### The pros and cons of the tDCS and TMS

Generally speaking, tDCS has several advantages compared with TMS, for example, tDCS is compact, portable, lower cost, and has small electrodes, which makes it more suitable for household and medical investigation. However, tDCS has several shortcomings, first, tDCS has a temporary skin reaction to the electrode, which might cause pain; second, the spatial accuracy of tDCS is worse than TMS (Gandiga *et al*., 2006); and third, tDCS is usually made by two electrodes on the scalp, and current formed between the reference electrode and the target electrode will interfere with the cortical excitability (Nitsche *et al*., 2007).

TMS is a safe means of physical therapy. Compared with drug therapy, it has a quick effect, a few side effects, and is not easy to become addicted. TMS is low cost for long-term treatment. Compared with other physical therapy, especially electroconvulsive therapy, it is safe, painless, and does not affect cognitive function. The main side effects of TMS are head discomfort, such as dizziness and headache. There are a few cases of epilepsy. The most common discomfort during treatment is local to the scalp. Most of these cases are mild and temporary. They usually fade after a week.

### tDCS of the DLPFC as a treatment for addiction

Preventing relapse is one major task in addiction management. Although the influence factors and the neurobiological mechanism are not fully clear, a great amount of evidence indicates that addiction relapse is largely affected by craving. The application of tDCS on the DLPFC to modulate craving brings up abundant results: in a randomized, double-blind, sham-controlled study, tDCS stimulation on both the left and the right DLPFC results in remarkably reduced cue-provoked craving (Fregni *et al*., 2008), and a cumulative effect of tDCS significantly attenuating smoking cue-induced craving has also been confirmed in another study (Boggio *et al*., 2009). Moreover, the smokers exhibited significantly decreased nicotine cravings and more active rejections to cigarette offers after tDCS stimulation on the right DLPFC than those with sham treatment (Fecteau *et al*., 2014). One study found that tDCS reduces the negative effect rather than cigarette craving and attention performances in overnight abstinent smokers, possibly due to the experiment design and withdrawal length differences (Xu *et al*., 2013). Another study reported that active prefrontal tDCS significantly decreased the rest craving state in the abstinent methamphetamine users, but increased craving if the stimulation is administrated during cue exposure (Shahbabaie *et al*., 2014). Participants who received active stimulation of tDCS showed reduced alcohol consumption, decreased craving, and improved cognitive performances (da Silva *et al*., 2013). In a study on alcohol addiction, 13 alcohol addicts were selected for double-blind real and sham tDCS stimulation with electrodes placed in the DLPFC (anode-left/cathode-right and anode-right/cathode-left), and alcohol-related videos were presented to the participants to induce alcohol cravings, and craving was measured by the Visual Analogue Scale before and after the simulation. The results showed that the real stimulation on the left side of the anode/right side of the cathode and the real stimulation on the right side of the anode/left side of the cathode significantly reduced craving compared to sham stimulation (Boggio *et al*., 2008). Alcohol craving was markedly decreased after tDCS to the left DLPFC, but was not influenced by tDCS to the right frontal gyrus, a region related to response inhibition, measured by the implicit association test (den Uyl *et al*., 2015). Klauss *et al*. conducted a study using 2 mA of stimulation in cocaine dependence with positive results (Klauss *et al*., 2018). The mechanism underlying the modulation is yet unclear, with few electrophysiological studies suggesting that tDCS modulate the activity or connectivity between the DLPFC and other prefrontal areas (Conti *et al*., 2014; da Silva *et al*., 2013; Nakamura-Palacios *et al*., 2012; Shahbabaie *et al*., 2018). A recent study investigated the combined effects of tDCS acting on the DLPFC and computerized cognitive addiction treatment (CCAT) on cue-induced craving and cognitive functioning in female patients living with methamphetamine use disorder. The investigators recruited 75 patients and randomly assigned them into three groups: a CCAT + tDCS group, a CCAT + sham tDCS group, and a control group. The first two groups underwent 20 sessions of cognitive training combined with 1.5 mA active/sham tDCS (20 min per session, five sessions per week) on the DLPFC and the control group underwent usual care. Cue-induced craving and cognitive function were tested at baseline, at week 2, and at the end of week 4. The results showed that CCAT + tDCS group significantly reduced cue-induced craving after 4 weeks of intervention. At the end of week 4, craving scores were significantly lower in the real CCAT + tDCS + group than in the CCAT + sham CCAT group and the control group. Compared to baseline, a significant improvement in the accuracy of the two back task was observed only in the CCAT + tDCS group at the end of week 4. Surprisingly, participants who received CCAT + active or sham tDCS did change their discounting, while participants in the control group showed more impulsivity over time. The study found a potential benefit of tDCS over DLPFC combined with CCAT to improve treatment outcomes in methamphetamine use disorder patients (Xu *et al*., 2021). In addition, a double-blind sham control study showed that tDCS on DLPFC had some effect on patients with cocaine use disorder over an extended treatment period (Gaudreault *et al*., 2021).

With dense connectivity to DLPFC, the fronto-parieto-temporal junction region demonstrated its usefulness in regulating smoking-related behavior and cue-induced heroin craving (Meng *et al*., 2014; Wang *et al*., 2016). (Table 1)

**Table 1:** Studies about tDCS of the DLPFC for addictive disorders.

Studies	*n*	Participants	Design	Number of sessions	Stimulation site	Polarity	Current (mA)	Effects	Side effects	Location
**DLPFC as the stimulation site**										
Fregi *et al*. (2008)	24	Smokers: >15 cigarettes per day; 1.5 h abstinence	Randomized, double-blinded, sham-controlled, crossover study	3 (1 anodal left; 1 anodal right; 1 sham)	Left and right DLPFC	A/C/S	2	Reduction of cue-induced craving after both active tDCS conditions; no effect on mood changes	Scalp burning, headache, itching, no difference between groups	10–20 EEG system
Xu *et al*. (2013)	24	Smokers: >15 cigarettes per day; 1.5 h abstinence	Single-blind, sham-controlled study	2 (1 Ac and 1 S)	Left DLPFC	A/S	2	No effect on cue-induced nicotine smoking following active tDCS; no effect on attention; mood improvement	Tingling, scalp burning, sleepiness	10–20 EEG system
Boggio *et al*. (2009)	27	Smokers: > 10 cigarettes per day; 1.5 h abstinence	Randomized, double-blinded, sham-controlled, crossover study	5	Left DLPFC	A/S	2	Reduction of cue-provoked craving and cigarette consumption	Scalp burning, headache, itching; no difference between groups	10–20 EEG system
Fecteau *et al*. (2014)	12	Light, moderate and heavy smokers	Randomized, double-blind, sham-controlled study	5	Left and right DLPFC	A/C/S	2	Reduction in number of cigarettes smokers lasting up to 4 days following active stimulation. No effect on risk task. Effect on Ultimatum task, rejection of cigarettes as an offer	Sleepiness, headache, pain	10–20 EEG system
Nakamura-Palacios *et al*. (2012)	49	Alcohol-dependent: detoxified; treatment-seekers; 7 days abstinence	Randomized, single-blind, sham-controlled study	2 (1 Ac and 1 S) while participants were exposed to visual cues	Left DLPFC	A/S	1	No effect on frontal activity as indexed by P3 in response to auditory alcohol related stimuli, except that in Lesch IV alcoholics showing increasing P3 amplitude after real tDCS	Itching and tingling reported for both sham and real stimulation	10–20 EEG system
Boggio *et al*. (2008)	13	Alcohol-dependent: detoxified; treatment seekers; >10 days abstinence	Randomized, double-blind, sham-controlled, cross-over study	3 (1 anodal left/cathodal right; 1 anodal right/cathodal left; 1 sham)	Left and right DLPFC	A/C/S	2	Reduction of craving by both active tDCS conditions	Discomfort, headache	10–20 EEG system
da Silva *et al*. (2013)	13	Alcohol-dependent: detoxified; in treatment; >10 days abstinence; in routine clinical treatment	Randomized, single-blind, sham-controlled study	5 (1/week)	Left DLPFC	A/S	2	No effects of real stimulation on relapse rate; reduction in craving for alcohol and in POMS depression score	NS	10–20 EEG system
den Uyl *et al*. (2015)	41	Heavy drinkers	Randomized, double-blind, sham-controlled study	3 (1 DLPFC; 1 IFG; 1 sham)	Left DLPF and IFG	A/S	1	Reduction of inclination toward drinking by real DLPFC stimulation. No effect of IFG stimulation. No effect of real stimulation on alcohol-related attentional biases	None	10–20 EEG system
Conti *et al*. (2014)	13	Crack-cocaine: average of 30 days of abstinence	Randomized, single-blind, sham-controlled study	5	Left and right DLPFC	A/C/S	2	Inhibitory effect on P3 drug-cued cortical activation by a single dose of active tDCS; increase after 5 sessions	Itching and tingling reported for both sham and real tDCS	10–20 EEG system
Gaudreault *et al*. (2021)	17	Cocaine inpatients	Randomized double-blind sham-controlled	2 (1 right anodal/left cathodal; 1 sham)	Left and right DLPFC	A/C/S	2	Reduction in craving induced by real stimulation	Tingling, burning, itching, and the experience of fatigue	10–20 EEG system
Shahbabaie *et al*. (2014)	32	Methamphetamine-dependent; abstinent	Randomized, double-blind, crossover sham-controlled study	2 (1 Ac and 1 S) at rest and during cue-inducing task	Right DLPFC	A/S	2	Reduction in spontaneous craving after 10 min stimulation induced by real stimulation; increase in cue-induced craving by real stimulation	Drowsiness, tingling, itching	10–20 EEG system
Shahbabaie *et al*. (2018)	15	Methamphetamine-dependent	Double-blind, sham-controlled crossover study	2 (1 anodal right/cathodal left; 1 sham)	Left and right DLPFC	A/S	2	Reduction in craving induced by real stimulation	NS	10–20 EEG system
Xu *et al*. (2021)	75	Methamphetamine-dependent; abstinent	Randomized, single-blind, sham controlled study	3 (1 CCAT + anodal right/cathodal left; 1 CCAT + sham;1 control)	Left and right DLPFC	A/C/S	1.5	Reduction in cue-induced craving and improvement in accuracy of two back tasks of real stimulation	Tingling and itching	10–20 EEG system
**Other stimulation sites**										
Wang *et al*. (2016)	20	Heroin-dependent; in treatment; 1.5–2 years abstinence	Randomized, single-blind, sham controlled study	2 (1 Ac and 1 S)	FPT	C/S	1.5	Significant difference between pre- and poststimulation cue-induced craving score following real stimulation	None	10–20 EEG system
Meng *et al*. (2014)	30	Smokers: >8 cigarettes per day	Randomized, double-blind, sham-controlled study	3 (2 Ac and 1 S)	FPT	C/S	1	Bilateral cathodal stimulation of the FPT areas significantly reduced the attention to smoking-related cues and daily cigarette consumption on the following day	Tingling, itching, pain	10–20 EEG system

A = anodal; Ac = active; C = cathodal; FPT = fronto-parietal-temporal areas; IFG = inferior frontal gyrus; NS = not specified; POMS = Profile of Mood States; S = sham.

### rTMS of the DLPFC as a treatment for addiction

rTMS has been approved by FDA for depression treatment, and is being clinically tested for an arsenal of psychiatric disorders, such as anxiety, schizophrenia, and obsessive-compulsive disorder. Two pioneering experiments uncovering the relationship between rTMS and subjective craving or consumption were first performed on smokers: HF rTMS of the left DLPFC significantly reduced cigarette smoking and significantly decreased nicotine craving (Eichhammer *et al*., 2003). Such an effect has been further confirmed by several other studies: for example, smokers exposed to smoking-related pictures followed a HF rTMS of the DLPFC remarkably reduced nicotine consumption or nicotinic craving (Amiaz *et al*., 2009). Recent studies have further shown that HF rTMS of the left DLPFC remarkably reduces heroin/cocaine/methamphetamine cue-induced craving in long-term addicts (Liang *et al*., 2018; Liu *et al*., 2017; Liu *et al*., 2019; Shen *et al*., 2016; Su *et al*., 2017; Terraneo *et al*., 2016). However, other research has failed to observe any effects of rTMS of DLPFC on alcohol craving or intake in alcohol-dependent participants (Del Felice *et al*., 2016; Höppner *et al*., 2011).

Most clinical rTMS studies related to substance use disorders use HF rTMS. HF rTMS is designed to increase the activity of frontal neural circuits, and the withdrawal phase of substance use disorders is marked by hypoactivity of frontal neural circuits, which is accociated with a weakening of executive control networks and a reduction in dopaminergic transmission. In clinical studies, HF rTMS acting on DLPFC has a cue-induced craving-reducing effect. Most studies adopt long-term stimulation (e.g. 20 stimulation sessions, once a day), and some studies use single sessions. Single sessions often produce inconsistent results. One study reported a significant reduction in craving in methamphetamine dependent individuals after a single session of stimulation using 10 Hz for both left and right DLPFC, while there was no change in the control condition (Liu *et al*., 2017). Simultaneously, a small sample of cocaine-dependent individuals undergoing a single session of the right DLPFC stimulation showed a decrease in craving, whereas stimulation of the left DLPFC showed no change (Camprodon *et al*., 2007). Another sham-controlled study found that a single session of 10 Hz rTMS acting on the left DLPFC significantly reduced craving in patients with methamphetamine substance use disorder (Su *et al*., 2017). HF rTMS applied to multiple sessions may have better and more reliable treatment outcomes for substance use disorders than single sessions. A meta-analysis examining single and multiple sessions of neuromodulation across all substance use disorder domains found that multiple sessions of neuromodulation were more effective in reducing craving compared to single sessions (Song *et al*., 2019). In fact, it has been shown that after the first treatment, there was no change in craving, while after five HF rTMS sessions there was a significant reduction in craving, whereas the sham group did not show the same effect (Su *et al*., 2017). In addition to reducing craving, studies have shown that long-term HF rTMS can improve several aspects of withdrawal symptoms, anxiety and depression scores, sleep quality, and cognition in patients with methamphetamine or heroin substance use disorder (Liang *et al*., 2018; Lin *et al*., 2019; Su *et al*., 2017). HF rTMS acting on the left DLPFC also reduced cue-evoked craving in patients with long-term heroin use disorder in a randomized, pseudo-stimulation-controlled crossover study in which researchers assessed participants’ cue-evoked craving at baseline, after the first day of treatment, and at the end of the fifth day of treatment. After the first treatment and after the fifth day of treatment, heroin use disorder patients showed a significant decrease in craving in both the first and fifth day of treatment (Shen *et al*., 2016). Thus, long-term rTMS may have an improving effect on many aspects for patients who live with substance use disorder, possibly due to altered frontal cortex plasticity. The iTBS is becoming increasingly popular in rTMS treatment, especially for treatment of major depression, due to its shorter stimulation time and higher efficiency compared to the traditional HF 10-Hz protocol (Blumberger *et al*., 2018). There have been no studies setting up the sham group iTBS to treat substance use disorders. A recent preliminary study using iTBS or 15-Hz intervention in patients with cocaine use disorder for 4 weeks and a twice-daily iTBS stimulation protocol in the first week found no significant differences in the treatment effects of the two treatment protocols on cocaine craving and usage amount. However, due to the lack of a sham group in this study, although there was a significant decrease in craving and usage amount in both treatment groups at 25 days posttreatment, the effect of time or a placebo effect could not be excluded (Sanna *et al*., 2019). This suggests that iTBS is as effective as 15 Hz in reducing cocaine craving and intake. Thus, the advantage of iTBS over 15 Hz is that the stimulation time is shorter and less intense, which makes iTBS more acceptable and tolerable for the patient, and more cost-effective for the clinician (Oberman *et al*., 2011). Another study found that a protocol of three daily iTBS interventions for 2 weeks significantly reduced cocaine intake as well as nicotine, alcohol, and tetrahydrocannabinol (Steele *et al*., 2019). Therefore, these studies provide preliminary evidence that iTBS is an effective and feasible treatment for patients with frequent cocaine use disorder.

The effects of low-frequency rTMS of the left DLPFC have not been adequately studied and are controversial. A single-blind, sham controlled crossover study, which recruited nontreatment-seeking methamphetamine users, found that 1 Hz rTMS intervention with left DLPFC increased cue-induced craving in the experimental group, but not in healthy controls, compared to the sham group (Li *et al*., 2013). In contrast, in a parallel, active control stimulation study with five 1-Hz rTMS stimulations on either the left or right DLPFC, cue-induced craving was significantly lower in patients with methamphetamine use disorder after both the first and the last stimulations compared to baseline at the time of treatment (Liu *et al*., 2017). The reason for the different results in these two studies may be that the participants in the former study were patients who were still using methamphetamine, and the participants in the latter were in rehab and had stopped using drugs in the past 2 months. Moreover, animal studies have shown that the accumulation of AMPA receptors is different at different stages of substance use disorders (Scheyer *et al*., 2016). In a recent double-blind, sham study, which recruited patients with methamphetamine use disorder, it was found that the 1 Hz rTMS intervention left DLPFC not only reduced cue-induced craving in the experimental group compared to the sham group, but also improved inhibitory control and reduced impulsivity in the experimental group (Yuan *et al*., 2020), and it is worth noting that the participants in this study were also in a drug rehabilitation facility. Similar to iTBS, cTBS (In the continuous theta burst stimulation paradigm (cTBS), a 40 s train of uninterrupted TBS is given (600 pulses)) is a shorter stimulation time protocol and has a stronger inhibitory effect on the cerebral cortex than 1 Hz (Huang *et al*., 2005). A preliminary sham-controlled study found that functional magnetic resonance imaging (fMRI) results showed a reduction activity in the insula, middle temporal gyrus, thalamus, and caudate nucleus regions after cTBS stimulation of mPFC in patients with chronic cocaine use disorder compared to the sham group, but the study showed no significant reduction in craving (Hanlon *et al*., 2015). In a larger, sham-controlled follow-up study, cTBS add-on the left mPFC-reduced activity in the striatum, ACC, and the parietal cortex, these regions associated with salience processing (ACC), attention/executive control (parietal cortex), and craving (striatum) in long-term cocaine dependents (Seeley *et al*., 2007). However, compared to the sham group, cTBS, while altering changes in brain activity, did not significantly alter cravings in patients with cocaine use disorder (Hanlon *et al*., 2017). In a follow-up study, a recent study added a cue response task before and after receiving a real or sham-stimulus to assess the state-dependent effects of rTMS. During the stimulation, participants were asked to think and describe the time of their last cocaine use, rather than simply resting. Compared to neutral cues, drug-related cues significantly increased functional connectivity in the mPFC, striatum, and areas associated with salience for cocaine users at baseline levels. Compared with the sham group, participants in the experimental group receiving cTBS had diminished frontal connectivity for drug-neutral cues, although there was no significant interaction with any regions of interest in the task, suggesting a general effect of cTBS on all brain regions (Kearney-Ramos *et al*., 2018). More recently, studies have also found that both HF and low-frequency rTMS over the left DLPFC are effective in reducing craving in people with heroin and methamphetamine use disorders, and the efficacy can be maintained for more than 60 days (Liu *et al*., 2020a; Liu *et al*., 2020b).

Besides many investigations of rTMS made on the left DLPFC, a small amount of rTMS have been conducted involving the right hemisphere; for example, one study observed that only the right DLPFC stimulation reduced the cocaine craving (Camprodon *et al*., 2007), and the craving score reduced significantly in patients receiving stimulation to either the right or the left side rTMS (Liu *et al*., 2017; Mishra, Praharaj *et al*., 2015). DLPFC connected brain regions could act as potential targets for TMS. A 10-Hz rTMS of the superior frontal gyrus, a brain region correlated with craving, induced elevated craving to a smoking cue but a reduced craving to a neutral cue (Rose *et al*., 2011). Although a great number of studies confirm that the craving is reduced in the addicts receiving rTMS compared with the sham controls, future studies are required to elucidate whether rTMS has a stable and persistent effect on improved craving (Table 2).

**Table 2:** Studies about rTMS of the DLPFC for addictive disorders.

Studies	*n*	Participants	Design	Number of sessions	Stimulation site	Frequency (Hz)	Percentage MT (%)	Total pulses per session	Effects	Side effects	Location
**DLPFC as the stimulation site**											
Eichhammer *et al*. (2003)	14	Nicotine dependent; motivated to quit smoking	Randomized, double-blind, sham-controlled, crossover study	4 (2 Ac; 2 S)	Left DLPFC	20	90	1000	Significant reduction in smoking in the rTMS group. No effect on craving	Mild headaches	5 cm
Amiaz *et al*. (2009)	48	Nicotine dependent; >20 cigarettes/day; motivated to quit smoking	Randomized, double-blind, sham-controlled study (active vs. sham rTMS/smoking-related vs. neutral) 10 daily sessions followed by a 4-week maintenance phase	10 daily sessions	Left DLPFC	10	100	1000	Significant reduction in cue-induced craving, cigarette smoking and dependence when participants received exposure to smoking cues followed by rTMS	NS	5 cm
Mishra *et al*. (2015)	20	Alcohol-dependent	Randomized, double-blind study	10 daily sessions	Left and right DLPFC	10	110	1000	Significant reduction in craving after the last rTMS session in both group	Nightmare and middle insomnia after the eighth session of one patient	NS
Del Felice *et al*. (2016)	17	Alcohol dependence	Randomized, sham-controlled study	2 (1 Ac and 1S)	Left DLPFC	10	100	1000	Significant improving inhibitory control task and selective attention and reduce depressive symptoms but not reduction in craving and alcohol intake	NS	10–20 EEG system
Camprodon *et al*. (2007)	6	Cocaine dependence	Randomized, cross-over study	2 (left or right side)	Left and right DLPFC	10	90	2000	Right but not left rTMS reduced craving	None	NS
Terraneo *et al*. (2016)	32	Cocaine dependence	Randomized, open-label study	2 (left or control)	Left DLPFC	15	100	2400	Reduction in craving by active rTMS	Mild scalp discomfort	MRI
Sanna *et al*. (2019)	25/22	Cocaine use disorder	NS	2 (2 Ac)	Bilateral PFC	iTBS/15	80/100	2400 and 600	Reduction in craving and the intake of cocaine in both groups	Mild scalp discomfort	NS
Steele *et al*. (2019)	19	Cocaine use disorder	Open-label	1 (1 Ac)	Left DLPFC	iTBS	100	600	Reduction both the amount and frequency of cocaine use	Occasional headaches	10–20 EEG system
Li *et al*. (2013)	10	Not-treatment seeking, methamphetamine users	Randomized, single-blind, sham-controlled, crossover study	2 (1 Ac and 1S)	Left DLPFC	1	NS	900	Increase in craving by active rTMS	Mild scalp discomfort	6 cm
Su *et al*. (2017)	30	Methamphetamine dependence	Randomized, double-blind, sham-controlled study	2 (1 Ac and 1S)	Left DLPFC	10	80	1200	No negative effects on cognitive function; reduction in craving for methamphetamine	Mild scalp discomfort	5 cm
Liu *et al*. (2017)	50	Methamphetamine dependence	Randomized, single-blind, sham-controlled study	5 (4 Ac and 1S)	Left and right DLPFC, P3	1 and 10	80	600 and 2000	Reduction in cue-induced craving by active rTMS	None	NS
Liang *et al*. (2018)	48	Methamphetamine dependence	Randomized, double-blind, sham-controlled study	2 (1 Ac and 1S)	Left DLPFC	10	80	2000	Reduction in cue-induced craving by active rTMS	Mild scalp discomfort	NS
Lin *et al*. (2019)	105	Methamphetamine dependence	Randomized, double-blind, sham-controlled study	3 (1 Ac, 1S and 1 C)	Left DLPFC	10	100	2000	Improved sleep quality, alleviated depression and anxiety state by active rTMS	Mild dizziness or scalp pain	10–20 EEG system
Liu *et al*. (2019)	90	Methamphetamine dependence	Randomized, single-blind study	2 (1 Ac; 1 C)	Left DLPFC	10	100	2000	Reduction in cue-induced craving by active rTMS	NS	5 cm
Yuan *et al*. (2020)	73	Methamphetamine dependence	Randomized, single-blind study	2 (1 Ac; 1S)	Left DLPFC	1	100	600	Reduction in cue-induced craving and improvement of impulse inhibition by active rTMS	NS	5 cm
Liu *et al*. (2020b)	188	Methamphetamine dependence	Randomized, single-blind study	3 (1 Ac; 1S; 1 C)	Left DLPFC	1 and 10	100	600 and 2000	Reduction in cue-induced craving by active rTMS	NS	10–20 EEG system
Shen *et al*. (2016)	20	Heroin dependence	Randomized, crossover, sham-controlled study	2 (1 Ac; 1S)	Left DLPFC	10	100	2000	Reduction in cue-induced craving by active rTMS	None	10–20 EEG system
Liu *et al*. (2020a)	118	Heroin dependence	Randomized, single-blind study	3 (1 Ac; 1S; 1 C)	Left DLPFC	1 and 10	100	600 and 2000	Reduction in cue-induced craving by active rTMS	Mild dizziness, headache, neck pain, insomnia, etc.	10–20 EEG system
**Other stimulation sites**											
Ross *et al*. (2011)	15	Smokers: > 20 cigarettes per day	Randomized, cross-over open-label study. At the beginning of each session, participants smoked a cigarette. 1 h later, they underwent rTMS concurrently during exposure to neutral/smoking cues/smoking a cigarette	3 (1 Hz SFG; 10 Hz SFG; 1 Hz motor cortex)	SFG or motor cortex (side not specified)	1 and 10	90	Greater number of pulses for the 10 Hz condition	Combination of smoking cues exposure and 10 HZ SFG rTMS increased craving	NS	10–20 EEG system

Ac = active; S = sham; C = control; MT = motor threshold; NS = not specified; SFG = superior frontal gyrus.

## Conclusion and Future Perspectives

In terms of addiction treatment, tDCS and rTMS are the two most commonly used noninvasive physical therapy methods. Both methods have the advantages of noninvasive and painless, and both can be combined with electroencephalogram (EEG), functional near-infrared spectroscopy, and other technical means for efficacy evaluation, and are therefore popular among researchers and physicians. These are widely used in psychology and clinical applications. However, there are also many differences between them. In comparison, tDCS is inexpensive and usually chooses DLPFC for anode or cathode electrical stimulation, for example, in one study, when electrodes were placed on the DLPFC (left side of the anode/right side of the cathode and the right side of the anode/left side of the cathode), alcohol addicts in the real stimulation group had significantly lower craving compared with the sham stimulation (Boggio *et al*., 2008). In studies related to addiction, researchers have located the DLPFC, which is located in the prefrontal cortex and is related to many functions. Previous studies have not clarified the direct link between this location and its effectiveness in improving addiction. Further research is needed to determine the validity of this location. While TMS is expensive and has different treatment targets depending on the purpose of treatment, researchers usually choose HF rTMS over the left DLPFC for addiction rehabilitation treatment.

In summary, in the studies related to addiction, researchers have largely localized this to the DLPFC, which is located in the prefrontal lobe and is associated with numerous functions. Previous studies indicate that HF rTMS of the left DLPFC and low-frequency rTMS over the right DLPFC may reduce cue-induced craving for methamphetamine (Liu *et al*., 2020b; Zhang *et al*., 2019). Different studies have confirmed the stimulation of this site benefits a decrease in craving, can overcome emotional problems, and promotes cognitive and executive functions due to substance addiction. For instance, recent studies expanded TMS targets for depression treatment to the ventrolateral prefrontal cortex, the ventromedial prefrontal cortex, frontopolar cortex, and dorsomedial prefrontal cortex (Downar and Daskalakis, 2013). These stimulation sites are potentially effective for treating addiction as well.

The following problems should be taken into consideration in future studies: (i) on which brain regions does NIBS achieve the optimal anti-craving effect? (ii) Although NIBS on DLPFC causes clinical effects, the underlying mechanisms are largely unknown, e.g. whether the effects are obtained by strengthening the left hemisphere or reducing the right hemisphere, or are they a summation of both? (iii) It would be interesting to combine NIBS on DLPFC with behavioral therapy in future management.
